# Phosphorylated-insulin growth factor I receptor (p-IGF1R) and metalloproteinase-3 (MMP3) expression in advanced gastrointestinal stromal tumors (GIST). A GEIS 19 study

**DOI:** 10.1186/s13569-016-0050-6

**Published:** 2016-06-29

**Authors:** Joan Maurel, Antonio López-Pousa, Silvia Calabuig, Silvia Bagué, Xavier Garcia del Muro, Xavier Sanjuan, Jordi Rubió-Casadevall, Miriam Cuatrecasas, Javier Martinez-Trufero, Carlos Horndler, Joaquin Fra, Claudia Valverde, Andrés Redondo, Andrés Poveda, Isabel Sevilla, Nuria Lainez, Michele Rubini, Xabier García-Albéniz, Javier Martín-Broto, Enrique de Alava

**Affiliations:** Department of Medical Oncology, Hospital Clinic, CIBERehd, Translational Genomics and Targeted Therapeutics in Solid Tumors (IDIBAPS), Barcelona, Spain; Department of Medical Oncology, Hospital Universitario Sant Pau, Barcelona, Spain; Molecular Oncology Laboratory, Fundación de Investigación del Hospital General Universitario de Valencia, Valencia, Spain; Pathology Department, Hospital Universitario Sant Pau, Barcelona, Spain; Department of Medical Oncology, Institut Català d’Oncologia L’Hospitalet, Barcelona, Spain; Pathology Department, Institut Català d’Oncologia L’Hospitalet, Barcelona, Spain; Department of Medical Oncology, Institut Català d’Oncologia de Girona, Girona, Spain; Pathology Department, Hospital Clínic, CIBERehd, IDIBAPS, Barcelona, Spain; Department of Medical Oncology, Hospital Universitario Miguel Servet, Saragossa, Spain; Pathology Department, Hospital Universitario Miguel Servet, Saragossa, Spain; Department of Medical Oncology, Hospital Central de Asturias, Oviedo, Spain; Department of Medical Oncology, Hospital Universitario Vall d’Hebron, Barcelona, Spain; Hospital Universitario La Paz, Madrid, Spain; Department of Medical Oncology, Fundación Instituto Valenciano de Oncología, Valencia, Spain; Department of Medical Oncology, Hospital Universitario Virgen de la Victoria y Regional de Málaga, Málaga, Spain; Department of Medical Oncology, Hospital de Navarra, Pamplona, Spain; Department of Experimental and Diagnostic Medicine, Department of Epidemiology, University of Ferrara (UNIFE), Emilia-Romagna, Italy; Harvard T.H. Chan School of Public Health, Boston, MA USA; Instituto de Biomedicina de Sevilla (IBiS), Hospital Universitario Virgen del Rocío/CSIC/Universidad de Sevilla, Seville, Spain; Pathology Department, Instituto de Biomedicina de Sevilla (IBiS), Hospital Universitario Virgen del Rocío/CSIC/Universidad de Sevilla, Seville, Spain

## Abstract

**Background:**

Most GISTs have mutations in KIT or PDGFRA. Patients with advanced GIST with KIT exon 9, PDGFRA mutation or WT for KIT and PDGFRA have a worse progression-free survival (PFS) compared to patients with KIT exon 11 mutated tumors. We evaluated the immunohistochemical (IHC) expression of p-IGF1R (Y1316) and MMP3 as predictors of PFS or overall survival (OS).

**Methods:**

Ninety-two advanced GIST patients included in GEIS-16 study with KIT and PDGFRA mutational information were examined for p-IGF1R (Y1316) and MMP3 expression in a tissue micro-array. To study activation of the IGF1R system, we have used an antibody (anti-pY1316) that specifically recognizes the active phosphorylated form of the IGF1R. DNA was extracted from paraffin-embedded tissues and intronic PCR primers were used to amplify exons 9, 11, 13 and 17 of KIT, 12 and 18 of PDGFRA. Bidirectional sequencing with specific primers was performed on a ABI3100 sequencer using the Big Dye Terminator v3.1 kit. Multivariate model was built using a stepwise automated variable selection approach with criterion to enter the variable in the model of p < 0.10 and criterion to keep the variable in the model of p < 0.05. PFS was computed as the date of imatinib initiation to progression or death. Overall survival was defined as the time from imatinib initiation to death.

**Results:**

Phospho-IGF1R was expressed only in 9 % (2/22) of cases without KIT mutation. MMP3 expression was detected in 2/5 patients (40 %) with PDGFRA mutation, 1/16 patients (6 %) with WT genotype and 7/71 patients (10 %) of KIT mutant patients. At univariate analysis KIT exon 11/13 mutation had better PFS than patients with exon 9 mutation, PDGFRA mutation or WT genotype (p = 0.021; HR: 0.46; 95 %CI (0.28–0.76). Less than 24 months disease free-interval (HR 24.2, 95 % CI 10.5–55.8), poor performance status (PS) (HR 6.3, 95 % CI 2.5–15.9), extension of disease; >1 organ (HR 1.89; 95 % CI 1.03–3.4) and genotype analysis (HR 0.57, 95 % CI 0.37–0.97) but not immunophenotype analysis (HR 1.53; 95 % CI 0.76–3.06) were the strongest prognostic factors for PFS in the multivariate analysis.

**Conclusions:**

Our results do not support p-IGF-1R and MMP3 evaluation in non-selected GIST patients but evaluation of this immunophenotype in WT and mutant PDGFR mutation in larger group of GIST patients, deserve merits.

## Background

Gastrointestinal stromal tumour (GIST) is the most common sarcoma of the gastrointestinal tract. Imatinib mesylate (IM), a receptor tyrosine-kinase (RTK) inhibitor active against *KIT* and *PDGFRA*, is the standard treatment for advanced GIST patients [1, 2]. Mutations in the KIT and PDGFRA oncogenes are identified in 85–90 % of patients with advanced GIST. Most mutations in advanced GIST are located in *KIT* exon 11 (68–75 %) but also in exons 9 (8–15 %), 13 and 17 (1 %) and PDGFRA homologous exons (2–4 %) [2–4].

A small subgroup of GIST patients (10–15 %) shows primary IM resistance (i.e. disease progression in the first 6 months of IM treatment). Unfortunately, 70–80 % of IM-sensitive patients acquire secondary resistance due to new IM-resistant KIT or PDGFRA mutations and KIT amplification [5]. Mutational analysis of these genes affects prognosis and responsiveness to tyrosine kinase inhibitors [2]. D842V PDGFRA (1 %) and RAS and BRAF (≤5 % of GIST) mutations, predicts primary IM resistance [6, 7].

Insulin-growth factor 1 receptor (IGF1R) is expressed in GIST patients [8, 9]. About 20–40 % of KIT/PDGFRA WT GIST patients show loss of function of the succinate dehydrogenase (SDH) including A, B, C, D complex which is associated to IGF1R expression [10, 11]. Although IGF1R expression is associated with a WT genotype, a very small subset of GIST SDHB-positive patients with mutations in KIT or PDGFRA (<1 % of all GIST) can also express IGF1R [11]. Recently IGF1R expression was found to be associated to lower response in advanced GIST but without affecting progression free survival or overall survival (OS) [12]. However, no previous studies have correlate IM efficacy and the activation of IGF1R (phospho-IGF1R). This aspect is important because phospho-IGF1R (p-IGF1R) expression does not correlate well with overall IGF1R expression [8]. MMP3 has been shown to be over-expressed (33-fold change) in a GIST-resistant (GIST882-R) cell line compared with the parental sensitive line [13].

Because p-IGF1R induce PI3K-AKT pathway activation and MMP3 can directly induce epithelial-mesenchymal transition [14], a widely known mechanisms of chemotherapy-resistance, we hypothesize that GIST patients with positive immunophenotype (either p-IGF-1R positive or MMP7 positive) can contribute to IM resistance. We selected patients with available tissue for biological analysis, from a cohort of advanced GIST patients treated with IM in 12 Spanish institutions included in the GEIS-16 study. The GEIS-16 study was a retrospective study to evaluate the role of metastatic surgical resection in GIST patients, sensitive to IM therapy [15].

## Patients and methods

### Study design

We selected patients from a cohort of advanced GIST patients treated with IM from January 2001 to December 2008 in 16 Spanish institutions included in the GEIS-16 study. Four institutions that participated in the GEIS-16 study did not participate in the GEIS-19 study. Only patients with available tissue for genotype and immunophenotype analysis were selected for the GEIS-19 study. Response rate was evaluated following RECIST criteria. The last patient status update was done in June 2015.

### Mutational analysis

DNA was isolated from 3 to 20 μm FFPET sections. After deparaffinization, DNA was extracted using the QIAamp^®^ DNA FFPE Tissue kit (Qiagen, Valencia, CA, USA) following the manufacturer’s instructions. Amplification of exons 9, 11, 13 and 17 of KIT and exons 12 and 18 of PDGFRA was carried out as previously described [14, 15]. Ten microliters of PCR products were visualized in ethidium-bromide-stained 2 % UltraPure agarose gel (Life Technologies, Paisley, Scotland) and photographed. Negative controls were included in each set of amplifications. Bidirectional sequencing with specific primers was performed on an AB I3130xL sequencer using the Big Dye Terminator v3.1 kit (Applied Biosystems, Inc, Foster City, CA). Sequencing analysis, version 5.2 software (Applied Biosystems) and the National Center for Bioinformatics Information blast tool (http://www.ncbi.nlm.nih.gov/BLAST/) were used to confirm the mutation sequences for KIT (ENSG00000157404) and PDGFRA (ENSG00000134853).

### Tissue microarray

Formalin-fixed paraffin embedded tissue samples of representative tumor regions from primary GISTs were collected for the preparation of 3 tissue microarrays. Briefly, three tissue cylinders with a diameter of 1.0 mm were punched out from morphologically representative areas of each donor tissue block and brought into a recipient paraffin block using a manual tissue arrayer.

### Immunohistochemistry and scoring

To study activation of the IGF1R system, we have used a primary antibody (anti-pY1316) that specifically recognizes the phosphorylated (active) form of the IGF1R (Generous gift of Dr. Rubini, Ferrara, Italy). Briefly, paraffin-embedded sections were deparaffinized in xylene and rehydrated in downgraded alcohols and distilled water. Heat-induced epitope retrieval and a high-pH buffer (for anti-p-IGF1R) and citrate buffer pH6 (for anti-MMP3) (both buffers from Ventana Medical Systems, Tucson, AR) were applied for 30 min before the primary antibody. Then, tissue microarrays were incubated with anti-pY1316 antibody (dilution 1/50), and with an anti-MMP3 antibody (Abcam #ab137659; dilution 1/50), followed by a specific secondary antibody using the DAB Map detection kit (Ventana Medical Systems, Tucson, AR). Sections were counterstained with hematoxylin and analyzed by light microscopy. Cases were scored as positive or negative. Cases were scored as positive when at least 1 % of cells showed cytoplasmic expression of the molecule under study (either p-IGF1R, or MMP3).

### Statistical analysis

Proportions are compared using Chi square test or Fisher’s test when appropriate. Means are compared using t test. Survival analyses are done using Kaplan–Meier estimates and Cox proportional hazards model. Progression-free survival is defined as the time from the date Imatinib was started to the date of progression or death whichever occurred first. Overall survival is defined as the time from the date Imatinib was started to the date of death. Multivariate models are built using two approaches: (1) Entering all the variables in the model. (2) Using a stepwise automated variable selection approach with criterion to enter the variable in the model of p < 0.10 and criterion to keep the variable in the model of p < 0.05.

## Results

**Fig. 1:**
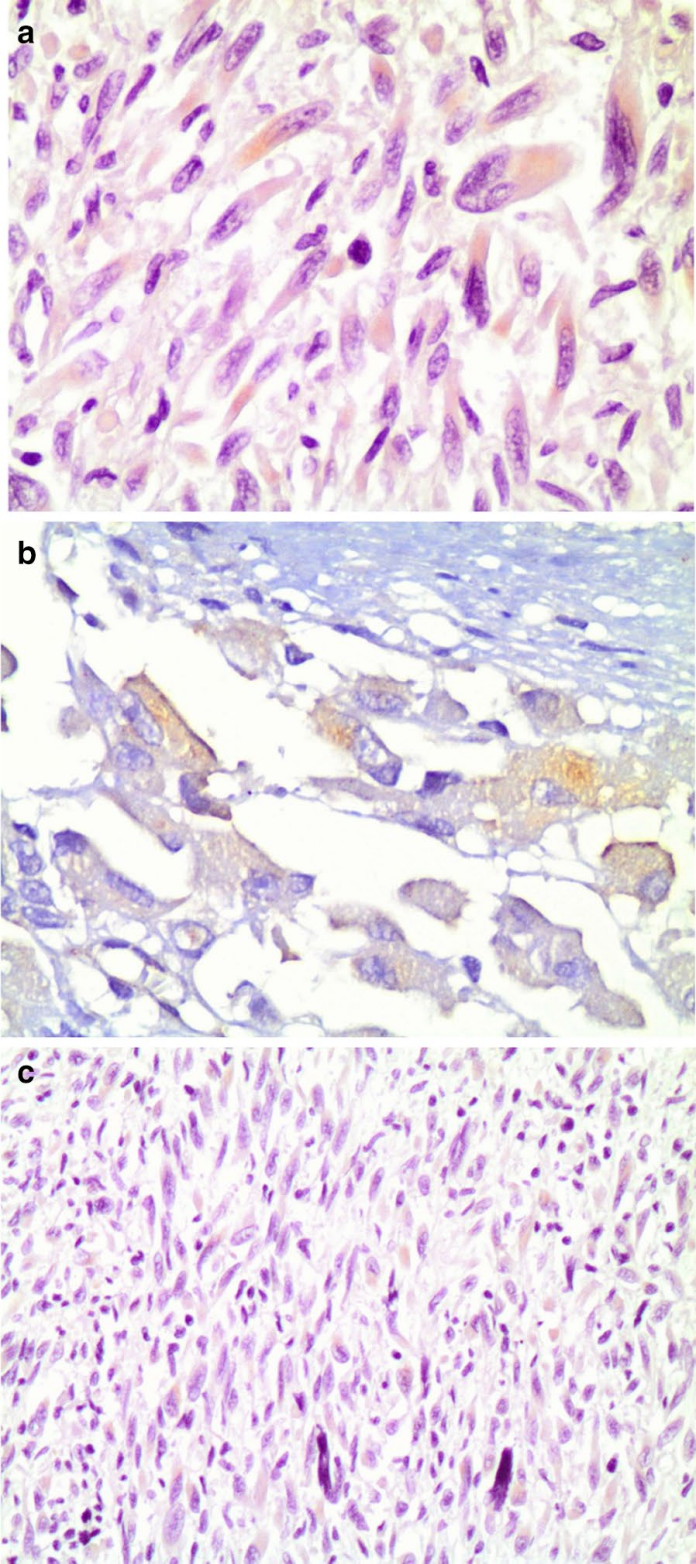
**a** Mild expression of MMP-3 is present in the cytoplasm of tumor cells, especially those with a higher degree of anaplasia. (×200) **b** Expression of p-IGF1R was seen in tumoral cells. (×400) **c** Expression of p-IGF1R was seen in tumoral cells. (×200)

Among 190 patients evaluated in the GEIS-16 study, 19 showed primary IM resistance (10 %). Paraffin-embedded tissue from primary tumours, were obtained from 92 untreated advanced GIST patients (46 % of the whole cohort of patients) for mutational analysis and tissue microarray construction (TMAs) in twelve Spanish Institutions. Eighty-eight patients were treated with 400 mg/day and 4 patients included in EORTC-ISG-AGITG phase III trial received 800 mg/day. Among 92 patients evaluated in GEIS-19 study 9 patients show primary IM resistance (10 %). Baseline characteristics of the patients according the immunophenotype are shown in Table 1. Seventy-one patients (78 %) had KIT mutations, 5 patients (5 %) had PDGFRA mutations and 16 patients (17 %) were WT for KIT and PDGFRA. Phospho-IGF1R was expressed only in 9 % (2/21) of cases without KIT mutation. MMP3 was expressed in 10 % of cases. MMP3 expression was detected in 2/5 patients (40 %) with PDGFRA mutation, 1/16 patients (6 %) with WT genotype and 7/71 patients (10 %) of KIT mutant patients. Positive immunophenotype, was mostly observed in WT and PDGFR genotypes (p = 0.006). Representative cases of p-IGF1R and MMP3 expression are shown in Fig. 1.

**Table 1:** Patients characteristics according phenotype

Characteristic	Phenotype− (N = 80)	Phenotype+ (n = 12)	p value
Female	33 (41 %)	8 (66 %)	0.13
Mean age (SD)	60.7 (18.8)	65.1 (12.3)	0.20
Metastatic status			0.8
1 site	61 (76 %)	10 (83 %)	
>1 site	19 (24 %)	2 (17 %)	
Primary site			0.4
Stomach	30 (37)	5 (42)	
Small bowel	33 (41)	2 (16)	
Other	17 (22)	5 (42)	
KIT/PDGFR status			*0.006*
KIT mutation	64 (79 %)	7 (58 %)	
PDGFR	2 (3 %)	3 (25 %)	
WT	14 (18 %)	2 (17 %)	
ECOG PS			0.79
0–1	72 (90 %)	11 (92 %)	
2	8 (10 %)	1 (8 %)	
Liver metastasis	48 (60 %)	7 (58 %)	0.91
Surgery of primary	74 (92 %)	11 (92 %)	0.91
Disease free interval			
<24 months	20 (25 %)	4 (33 %)	0.5
>24 months	60 (75 %)	8 (67 %)	
Mean LDH (SD)	332.1 (160.8)	482.4 (429.8)	0.39
Mean leucocytes (SD)	7.1 (3)	8.1 (5.1)	0.56
Mean albumin (SD)	40.9 (7.1)	39.6 (6.1)	0.62

*SD* standard deviation

**Fig. 2:**
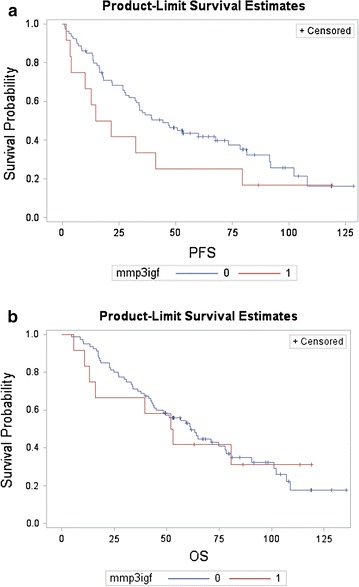
Cumulative survival curves for patients according MMP3/p-IGF-1R expression. **a** Progression-free survival. Log-rank test, 1.39, p = 0.34. **b** Overall survival. Log-rank test, 1.53, p = 0.23

Patients with mutations in KIT exon 11/13 showed higher response rate to IM (77 %) than patients with mutations in KIT exon 9 (25 %), PDGFRA (0 %) or wild-type (53 %) genotype (p < 0.0001). Response rate was observed in 4/11 (36 %) patients with MMP3 or p-IGF1R expression (1 patient was non-evaluable for response) vs 56/78 (71 %) in GIST patients without MMP3 or p-IGF1R expression (2 patients were non-evaluable for response) (p = 0.025). At univariate analysis KIT exons 11/13 had better PFS than patients with exon 9, PDGFRA mutation or WT genotype [p = 0.037; HR: 0.57; (95 % CI 0.33–0.97)]. Patients with MMP3 or p-IGF1R expression have non-significant poor PFS [14.1 months 95 % CI (0–29.8)] than patients without either p-IGF1R or MMP3 expression [37.1 months 95 % CI (25.3–48.9)] (p = 0.33) (Fig. 2a. Disease free-interval, performance status (PS), extension of disease and genotype but not immunophenotype (p-IGF1R or MMP3) were the strongest prognostic factors for PFS in the multivariate analysis. There were also no differences in survival according MMP3 or p-IGF1R expression (Fig. 2b). For OS only performance status, disease free-interval, surgery of primary tumor and number of metastatic sites remain significant (Table 2).

**Table 2:** Univariate and multivariate analysis

	Univariate (PFS)		Univariate (OS)		Multivariate (PFS)		Multivariate (OS)	
	HR (95 % CI)	p value	HR (95 % CI)	p value	HR (95 % CI)	p value	HR (95 % CI)	p value
Group								
MMP3 and pIGF1R−	Reference		Reference		Reference		Reference	
MMP3 or pIGF1R+	1.39 (0.71–2.72)	0.34	1.10 (0.52–2.17)	0.95	1.53 (0.76–3.06)	0.23	1.67 (0.77–3.65)	0.20
Albumin >median	0.73 (0.44–1.23)	0.24	0.82 (0.47–1.44)	0.49				
Leucocytes >median	1.16 (0.73–1.85)	0.53	1.50 (0.90–2.49)	0.12				
LDH >median	1.36 (0.84–2.21)	0.21	1.50 (0.89–2.55)	0.13				
Age	0.99 (0.97–1.00)	0.33	0.99 (0.98–1.01)	0.51				
Kit mutation								
Exon 11/13	0.46 (0.28–0.76)	0.021	0.71 (0.41–1.21)	0.21	0.57 (0.33–0.97)	*0.037*		
Other	Reference		Reference					
ECOG								
PS 0	Reference		Reference		Reference		Reference	
PS 1	1.13 (0.67–1.8)	0.65	1.17 (0.66–2.07)	0.59	1.74 (0.99–3.07)	*0.055*	2.00 (1.08–3.67)	*0.026*
PS > 1	1.64 (0.73–3.68)	0.23	2.05 (0.88–4.76)	0.095	4.45 (1.85–10.76)	*0.0009*	5.25 (2.08–13.26)	*0.0005*
Female	1.02 (0.64–1.63)	0.92	1.01 (0.61–1.67)	0.96				
Primary site								
Stomach	1.61 (1.00–2.59)	*0.050*	1.36 (0.82–2.27)	0.24				
Others	Reference		Reference					
Extension of disease								Ta
1 metastatic organ	Reference		Reference		Reference		Reference	
2 or more metastatic organs	2.59 (1.52–4.42)	*0.0005*	2.56 (1.46–4.52)	*0.0011*	1.84 (1.03–3.27)	*0.039*	1.93 (1.03–3.62)	*0.041*
Surgery primary tumor								
Yes	Reference		Reference					
No	1.19 (0.51–2.75)		0.75 (0.27–2.08)	0.59			0.26 (0.09–0.76)	*0.014*
Disease-free interval		0.69						
<24 months	13.4 (6.93–25.77)	*<0.0001*	6.61 (3.72–11.79)	*<0.0001*	18.87 (8.54–41.73)	*<0.0001*	16.73 (7.97–35.13)	*<0.0001*
≥24 months	Reference		Reference		Reference		Reference	

## Discussion

Our main findings reveal that, the proposed immunophenotype (p-IGF1R or MMP3 positive) correlates with poor response rate and a worse but statistically non-significant progression-free survival, after adjustment of all critical variables in the multivariate analysis.

IGF1R is expressed in a subset of GIST patients without KIT and PDGFRA mutations [10, 11]. We have confirmed that IGF1R activation is a rare event in KIT mutant patients but, although with a low frequency, this receptor is activated in GIST patients carrying PDGFRA mutations or WT genotype. MMP3 is expressed in less than 10 % of KIT mutant and WT genotype in advanced GIST patients and in 40 % of PDGFRA mutant patients [16]. Although our data is limited to five patients, it could have clinical implications, because new drugs with potential activity in PDGFRA patients such as crenolanib [17] could be inactive in PDGFRA mutant patients that express MMP3.

We are tempted to speculate that in a small subset of patients with GIST with KIT mutations (10 %) and an important subset of WT genotype and PDGFRA mutations (21 %) our proposed immunophenotype bypass KIT signaling. It has been previously published that GIST patients with KIT mutation express p-STAT3 and p-AKT more intensely than patients with PDGFRA mutation [18]. Because MMP3 thought RAC1b can activate NF-kB and cyclin D1 but not AKT and STAT3 [19] and IGF1R not only activates AKT but also MEK/ERK pathway, our proposed immunophenotype may confer KIT-independent IM resistance, specially, in the subset of WT or PDGFR mutant GIST patients.

Our study has several limitations. First the data comes from a retrospective cohort of patients and therefore PFS are more subject to investigator interpretation. Second, the phenotype implicates only 12 % of all the analyzed GIST patients. Third our cohort included a limited number of patients. Four, the percentage of WT KIT/PDGFR patients is slightly higher than in other published series. We cannot rule out that other more sensitive methods such as high-resolution melting analysis, could decrease the number of WT GIST patients. Finally, other RTK such as MET or FGFR3 that has been implicated recently in primary and secondary IM resistance, has not been evaluated [20, 21]. Despite of it, the multicenter nature of the study and the long follow-up (that include the genotype and all the important clinical variables) supports the strength of our conclusions.
